# Genome-wide identification of PSKR genes in wheat and differential expression under abiotic stress conditions

**DOI:** 10.3389/fpls.2025.1582433

**Published:** 2025-09-12

**Authors:** Zhenbing Ma, Jingren Wei, Jia Zheng, Jian Su, Dong Zhao

**Affiliations:** ^1^ Wuliangye Yibin Co., Ltd, Yibin, China; ^2^ College of Biological Engineering, Sichuan University of Science & Engineering, Yibin, China; ^3^ State Key Laboratory for Crop Stress Resistance and High-Efficiency Production, College of Plant Protection, Northwest A&F University, Xianyang, Shaanxi, China

**Keywords:** wheat, TaPSKR genes, abiotic stress, expression profile, qRT-PCR

## Abstract

Phytosulfokine (PSK) is an important plant growth factor regulating plant stress response, *PSKR* gene family plays an important role in the PSK signaling pathway, but there are few reports in wheat (*Triticum aestivum L.*). In this study, 149 *TaPSKR* genes were identified by using hidden Markov models (HMMs) and sequence homology, and their evolutionary relationship, structural characteristics and stress regulation mechanism were systematically analyzed. Our results indicated that genes were unevenly distributed across 21 chromosomes of the wheat genome. Phylogenetic tree and conserved motif analysis revealed that the gene motifs, domains, and structures were relatively conserved, and 75.2% of TaPSKR genes clustered in one clade with monocotyledons. Chromosome distribution showed that genes were enriched in chromosomes 6A (20.13%), 6B (16.11%) and 6D (18.79%). A total of 6,463 cis-acting elements were discovered in the promoter regions of PSKR genes, indicating that these genes are regulated by various developmental, environmental, and hormonal factors, as well as light responses. Expression profile results demonstrated that these genes were influenced by various abiotic stressors, such as PEG6000, salt, cold, heat, and different hormones, including gibberellic acid, jasmonic acid, abscisic acid, 6-benzylaminopurine, and salicylic acid. Additionally, quantitative real-time PCR was performed to further confirm the transcriptomic data. TaPSKR genes may participated in abiotic stress response and phytohormone regulation pathway, which provided key gene resources and theoretical basis for the analysis of PSKR signaling pathway and molecular breeding for stress resistance in wheat.

## 1 Introduction

The escalating threats of rising temperatures and water scarcity are significantly impacting crop productivity worldwide. Due to their inability to escape like animals, plants have evolved numerous mechanisms to adapt to the dynamic environment, thus guaranteeing survival under unfavorable conditions. Plants respond to biotic and abiotic stressors through signal perception, physical adaption, and molecular and cellular changes (Ahuja et al., 2010; Takahashi and Shinozaki, 2019). An increasing number of studies have investigated peptide hormones, such as phytosulfokine (PSK), as a central component of cell to cell communication that plays an important role in regulating plant growth and reproduction (Sauter, 2015; Hohmann et al., 2017; Godel-Je˛drychowska et al., 2019).

PSK, which is a disulfated pentapeptide, is considered a crucial plant growth factor. It was first identified in *Asparagus officinalis* mesophyll cells grown in a culture medium (Matsubayashi and Sakagami, 1996). PSK, which is encoded by the *PSK* gene family, is composed of five residues, including two sulfated tyrosines (Tyr (SO3H)-IleTyr (SO3H)-Thr-Gln) (Yang et al., 2001; Matsubayashi et al., 2006; Kutschmar et al., 2009). PSK is processed from an ~80-amino acid precursor protein and matured through proteolytic cleavage and post-translational sulfonation of its tyrosine residues (Srivastava et al., 2008). Tyrosine sulfation occurs in the trans-Golgi network, is catalyzed by a tyrosylprotein sulfotransferase, and is further cleaved by cytoplasm-localized subtilisin-like serine proteases (Kutschmar et al., 2009). Subsequently, mature PSK peptides are perceived by membrane-bound receptors (PSKRs), which were first isolated from *Daucus carrota*, and they are evolutionarily conserved in different species (Hanai et al., 2000; Kaufmann and Sauter, 2019). PSKRs are localized at the plasma membrane and belong to the canonical leucine-rich repeat receptor kinases (LRR-RK) family, which contains extracellular LRRs and a PSK-binding domain, a transmembrane domain, an intercellular kinase domain, and an island domain (Kwezi et al., 2011). A total of 2, 2, and 15 potential PSK receptors have been predicted in *Arabidopsis*, tomato, and rice, respectively (Matsubayashi et al., 2006; Amano et al., 2007; Zhang et al., 2018; Nagar et al., 2020).

In plants, PSK participates in growth and development regulation, including callus formation, cell proliferation, sexual reproduction, lateral root development, and biotic and abiotic stress responses (Matsubayashi and Sakagami, 1996; Matsubayashi et al., 1999; Stührwohldt et al., 2011; Sauter, 2015). In *Arabidopsis*, PSK is induced by wounding, and the *PSKR1* mutant inhibits callus formation, demonstrating that signaling through *PSKR1* is essential for cell proliferation (Matsubayashi et al., 2006; Loivama et al., 2010). Additionally, PSK-α positively regulates root growth, and the knockout of any PSKR results in short roots due to reduced root cell length (Kutschmar et al., 2009). Recent publications have shown that PSK signaling pathways fine-tune plant growth by acting together with other phytohormones (Rodiuc et al., 2016). For example, the *PSKR1* mutant is sensitive to the hemibiotrophic bacterial pathogen *Pseudomonas syringae* but insensitive to the necrotrophic fungal pathogen *Alternaria brassicicola*, suggesting that PSK has an antagonistic effect on salicylic acid (SA)-mediated defense responses (Mosher et al., 2013). In addition, PSK suppresses ethylene (ET) synthesis, regulates copper homeostasis, and stimulates non-embryogenic proliferation in cooperation with auxin (Eun et al., 2003). In *Arabidopsis*, ectopic expression of *OsPSKR15* from rice positively regulated drought tolerance by controlling stomatal movements in reactive oxygen species (ROS)-dependent abscisic acid (ABA) signaling (Nagar et al., 2022). *OsPSKR* has mutual antagonism with *OsPEPR1* signaling, thereby modulating the transition of defense signals to growth signals during wounding (Chitthavalli et al., 2024). In tomato, the PSK-PSKR signaling pathway triggers auxin-dependent immune responses against *Botrytis cinerea* by inducing intracellular Ca^2+^ release (Zhang et al., 2018). PSK is induced by drought and regulates flower drop (Reichardt et al., 2020).

Thus far, studies on the PSK-PSKR signaling pathway have mainly focused on *Arabidopsis* and rice. Wheat, one of the most important food crops, has a higher salt tolerance than rice. However, in wheat, there are few reports of the *PSKR* gene family, which plays an important role in the PSK signaling pathway and regulates plant growth and development and biotic and abiotic stress responses. In this study, the *PSKR* gene family was identified in wheat based on the latest genome sequence. We performed genome-wide characterization of PSKRs in wheat; determined the phylogenetic relationships between PSKRs in wheat, *Arabidopsis*, and rice; and identified the conserved motifs in wheat PSKRs and the cis-acting elements in the 2000-bp promoter of wheat *PSKR* genes. In addition, we determined the *PSKR* gene expression profile under NaCl, heat, cold and ABA treatment. Our results are expected to provide a basis for further revealing the function and molecular mechanism of abiotic stress tolerance of *TaPSKR* genes.

## 2 Materials and method

### 2.1 Plant material growth and abiotic stress conditions

Wheat (*T. aestivum*) cultivar ‘Fielder’ seedlings were grown in a growth chamber (white fluorescent tubes, 200 - 300 μmol m^−2^ s^−1^) at maintaining 14°C/12°C day/night temperatures (16/8 h photoperiod) at 60% humidity or in the field (natural long-day conditions). Seedlings at the 3-leaf stage were treated with 200 mM NaCl, 4°C, 24°C, and 100 µM ABA, respectively. All 3 leaves from at least 5 plants were separately collected 0, 1, 3, 6, 12, 24, and 48 h after treatment. All leaf samples were flash frozen in liquid nitrogen and ground in a mortar for RNA isolation.

### 2.2 Identification and characterization of *TaPSKR* genes in wheat

To identify the potential *PSKR* genes in *T. aestivum*, hidden Markov models (HMMs) and sequence homology were used. Two *AtPSKR* genes (*At2g02220* and *At5g53890*) and fifteen *OsPSKR* genes were retrieved from TAIR (https://www.arabidopsis.org/) and the Rice Genome Annotation Project (https://rice.uga.edu/index.shtml), respectively, and used to search for PSKR members in *T. aestivum* (IWGSC.57) using HMMER (hmmer.org). The protein sequences were downloaded from Ensembl plants (release 59). TBtools-II was used to filter genes dissimilar to other species with PSKR proteins (Chen et al., 2023).

### 2.3 Phylogenetic analysis and chromosomal localization of *TaPSKR* genes

A phylogenetic tree of *PSKR* genes in wheat, *Arabidopsis*, and rice was constructed using the maximum likelihood (ML) method with 1000 bootstrap replicates and combined with the protein domain structure and gene structure using iTOL (https://itol.embl.de/). The *TaPSKR* genes of wheat were mapped to 21 chromosomes using genome data from TBtools-II (Chen et al., 2023).

### 2.4 Gene structure, conserved motifs, and cis-element analysis of *PSKR* genes

Gene structure annotation was performed using the GFF3 file of the wheat genome and visualized using TBtools v2.025 (Chen et al., 2023). MEME (https://meme-suite.org/meme/) was used to identify the conserved motifs of TaPSKR proteins. The potential cis-elements predicted in the 2-kb region upstream of the start codon of *TaPSKR* genes in the wheat genome were used in promoter sequence analysis with the PlantCARE database (https://bioinformatics.psb.ugent.be/webtools/plantcare/html/).

### 2.5 Expression profile analysis of *TaPSKR* genes under different abiotic stress and phytohormone treatments

The transcriptome data of *TaPSKR* genes in wheat under different abiotic stress and phytohormone treatments were downloaded from the WheatOmics 1.0 database (http://202.194.139.32/). A heat map was constructed using TBtools-II (Chen et al., 2023).

### 2.6 Quantitative real-time PCR analysis

RNA was extracted from the leaves of both the control and treatment groups. Quantitative real-time PCR (qRT-PCR) was performed to analyze the expression of 13 randomly selected *TaPSKR* genes in wheat under 200 mM NaCl, 100 µM ABA, cold (4°C), and heat (24°C) treatments. The total qRT-PCR volume was 20 µL: 10 µL of SYBR Premix Ex Taq (RR820 Takara, China), 0.5 µL of each primer (primers are listed in **Supplementary Table S1**), 1 µL of cDNA template, and 8 µL of ddH_2_O. PCR was performed as follows: initial pre-denaturation at 95°C for 2 min; 40 cycles of 95°C for 10 s, annealing at 56°C for 30 s, and extension at 72°C for 15 s. The experimental results were normalized using the 2^−▵▵Ct^ method.

## 3 Results

### 3.1 Identification of *PSKR* genes in wheat

In this paper, we used the HMM and BLASTp approaches. A total of 149 *PSKR* genes, named *TaPSKR1* to *TaPSKR149*, were identified in the wheat genome based on 2 *Arabidopsis thaliana* and 15 *Oryza sativa* gene sequence searches (**Supplementary Table S2**). We then analyzed detailed information on the 149 *TaPSKR* genes. Consistent with previous studies, transmembrane domain prediction showed that all proteins had a single membrane spanning domain (https://services.healthtech.dtu.dk/services/DeepTMHMM-1.0/; Matsubayashi et al., 2006; Hallgren et al., 2022). The number of amino acids in these proteins ranged from 155 (*TraesCS2A02G550500/TaPSKR9*) to 1205 (*TraesCS6A02G129000/TaPSKR88*), with an average of 789 amino acids. The isoelectric point (pI) ranged from 4.88 (TraesCS6B02G154700/TaPSKR102) to 9.66 (TraesCS1B02G193000/TaPSKR3), with an average of 6.52. The molecular weight (MW) ranged from 16.76 (TraesCS6A02G128600/TaPSKR79) to 130.18 KD (TraesCS6A02G129000/TaPSKR80), with an average of 81.2 KD. In addition, we exported the cDNA, coding, and protein sequences.

### 3.2 Phylogenetic, motif distribution, conserved motif, and exon-intron structure analysis of wheat PSKR proteins

**Figure 1:**
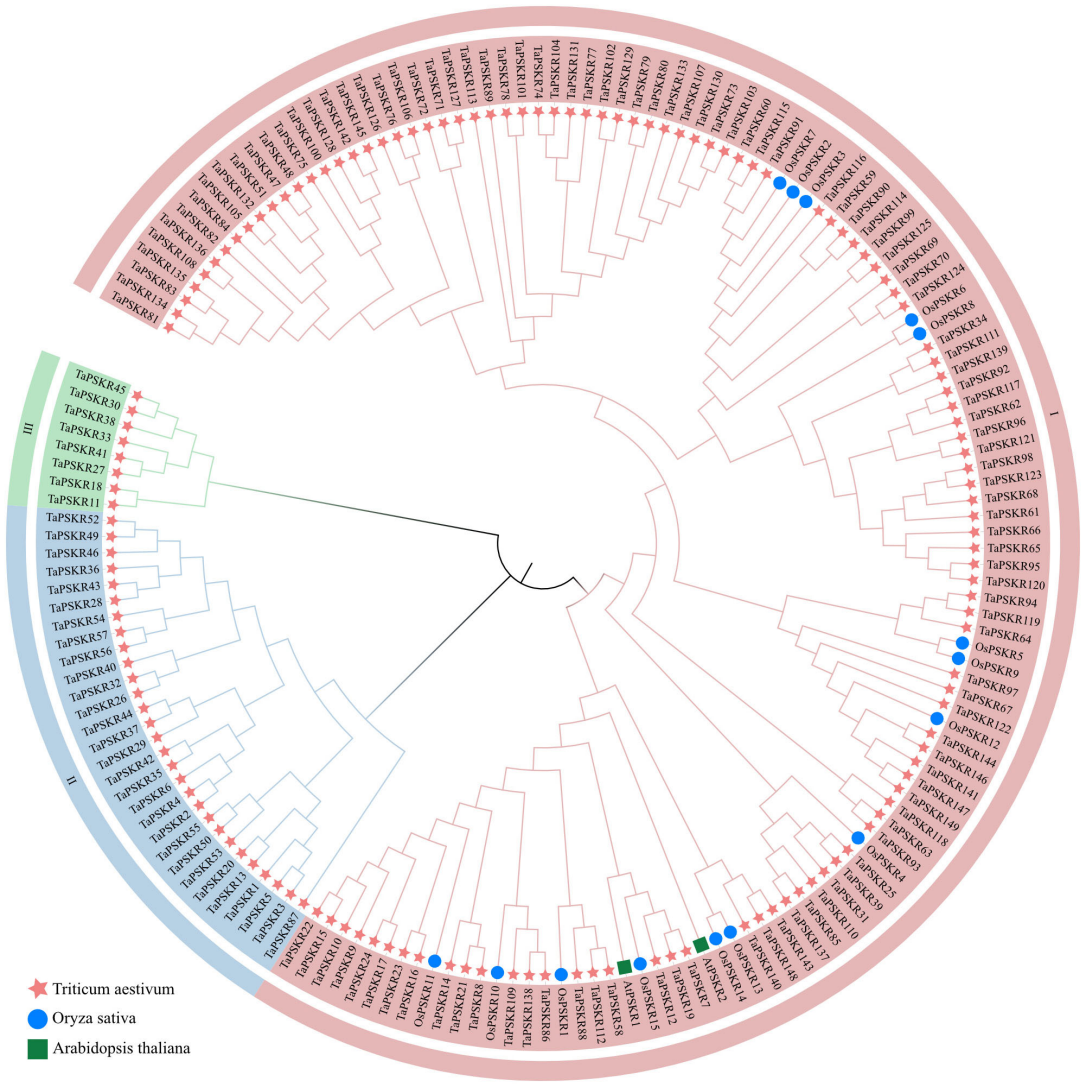
Phylogenetic analysis of PSKR proteins in wheat, *Arabidopsis*, and rice. The phylogenetic tree was constructed using the neighbor-joining method with 1000 bootstrap replications. In the tree, the TaPSKR proteins were divided into 3 subgroups, which are represented by different colored clusters within the tree.

To investigate the relationships between these PSKR proteins, we constructed a phylogenetic tree using the protein sequence of 149 *TaPSKR*, 2 *AtPSKR*, and 15 *OsPSKR* genes (**Figure 1**). These *TaPSKR* genes were divided into 3 subfamilies using tree-based distances in TreeCluster (https://github.com/niemasd/TreeCluster; Balaban et al., 2019). Significant variations were observed in the number of genes within each branch. The most members were in branch I, which contained 112 *TaPSKR* genes (accounting for 75.2% of the total). Notably, all *AtPSKR*s and *OsPSKR*s were distributed in branch I, highlighting the evolutionary conservation of *PSKR* genes across species in branch I. In branch I, most TaPSKR proteins formed a cluster with OsPSKR proteins, separate from the orthologs in *Arabidopsis*, suggesting that *TaPSKR* genes have more sequence similarity with monocotyledons than dicotyledons.

### 3.3 Gene structure and conserved motifs in *TaPSKR* genes

**Figure 2:**
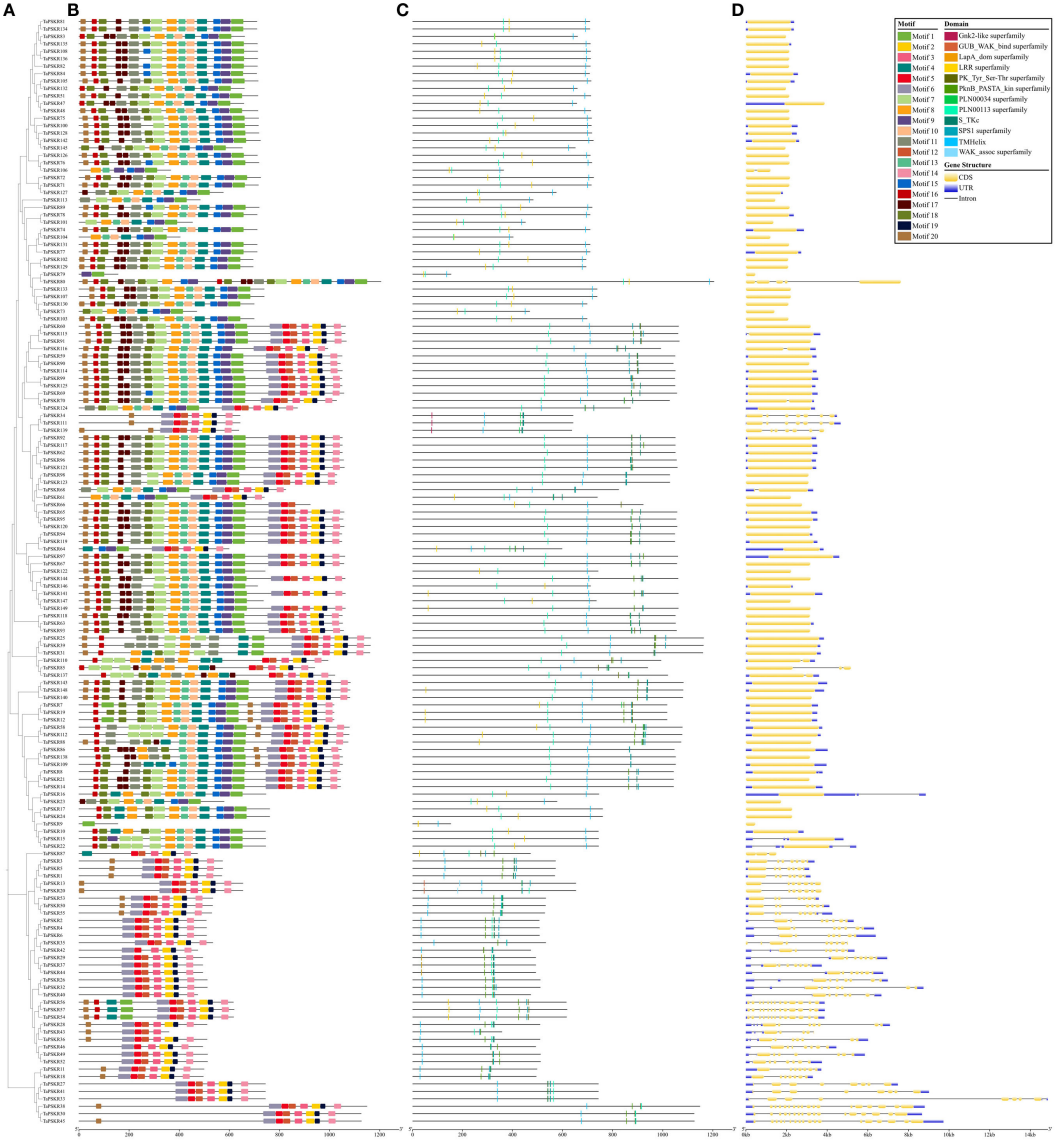
Gene structure and conserved domain organization of *PSKR* genes in wheat. **(A)** Their phylogenetic relationships based on the neighbor-joining method. **(B)** Motif distribution of the TaPSKR proteins. Different motifs (1-20) are indicated by different colors. **(C)** Conserved domain organization in TaPSKR proteins. **(D)** Exon–intron structures of *TaPSKR* genes.

To understand the structural composition of the *TaPSKR* gene family, a phylogenetic tree was constructed to elucidate the relationships among all *TaPSKR* genes (**Figure 2A**). Based on tree topology, we further analyzed the conserved motifs, domains, and exon-intron structure features of *TaPSKR* genes. A total of 20 conserved motifs (named motifs 1-20) were generated for all *TaPSKR* genes using the MEME tool (**Figure 2B**; **Supplementary Table S3**). The conserved motifs ranged from 1 (*TraesCS2A02G550500/TaPSKR9*) to 20, and 42 TaPSKR proteins contained less than 10 motifs. A total of 58 TaPSKR proteins contained 10–19 motifs, and 49 TaPSKR proteins contained 20 conserved motifs. Motifs 2, 5, 6, 12, 14, 15, and 16 were located within the C-terminal, and motifs 10, 13, 17, 18, 19, and 20 were located within the N-terminal. Consistent with the gene structure distribution, TaPSKR proteins containing a similar conserved motif were clustered together in the phylogenetic tree (**Figure 2B**). Consistent with other LRR-RLKs, all TaPSKR proteins contained a single transmembrane domain (TMHilex) and diverse kinase domain types, such as the Ser-Thr kinase superfamily, PknB_PASTA kinase superfamily (Matsubayashi et al., 2006; Burastero et al., 2022), SPS1 superfamily, and WAK-associated superfamily. In addition, other domains, such as the LRR superfamily, LapA domain superfamily response to heat shock, Gnk2-like superfamily response to abiotic stress (Serrazina et al., 2022), GUB_WAK_bind superfamily response to salt stress (Tang et al., 2022), and the PLN0034 and PLN00113 superfamily, were detected in these proteins (**Figure 2C**; **Supplementary Table S4**). The gene structure displayed significant diversity among the *TaPSKR* genes; the exon number ranged from 1 to 18, with the majority containing only 1 exon (98, 65.77%), 6 containing 2 exons, *TaPSKR85* and *TaPSKR87* containing 3 exons, *TaPSKR38* and *TaPSKR45* containing 18 exons, and the remainder containing between 3 and 17 exons (**Figure 2D**; **Supplementary Table S5**). Diversity variations likely reflect the different functional roles and evolutionary trajectories of these genes, suggesting diverse regulatory mechanisms within the *TaPSKR* gene family related to plant growth and development. Generally, the same subgroup in the phylogenetic tree shared a similar exon-intron structure.

### 3.4 Chromosomal location analysis of *TaPSKR* genes

**Figure 3:**
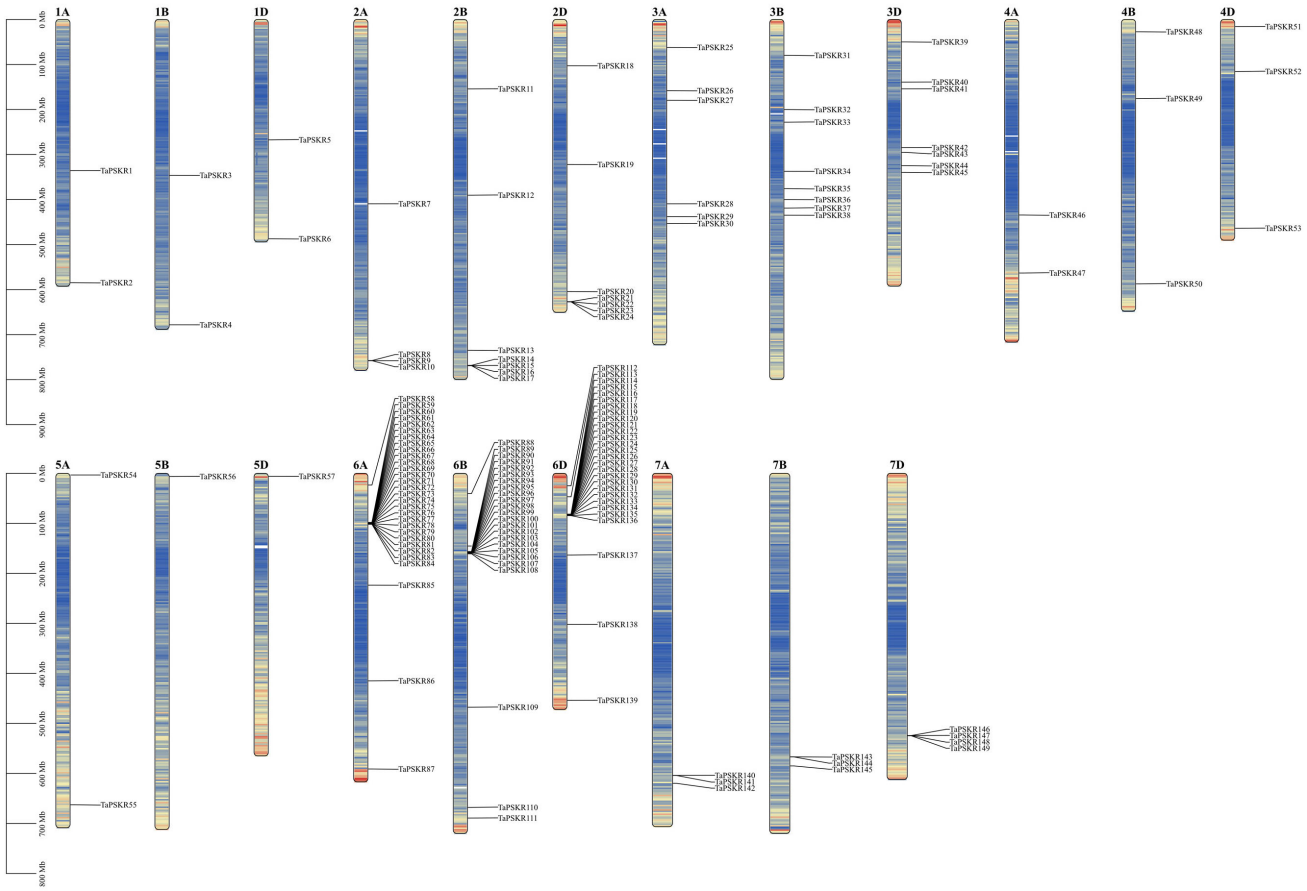
Chromosomal distribution of *TaPSKR* gene copies. Only chromosomes containing *TaPSKR* genes are represented.

To analyze the relative position of each *TaPSKR* gene copy on the chromosome, we labeled their physical placements on wheat A, B, and D chromosomes. As shown in **Figure 3**, the *TaPSKR* genes were mapped on the 21 chromosomes with a significantly uneven distribution. Only 1 gene (5.26%) was located on chromosomes 5B and 5D, and most of the *TaPSKR* genes were mapped on chromosome 6A (30/20.13%), followed by chromosome 6B (24/16.11%) and chromosome 6D (28/18.79%).

### 3.5 Prediction of cis-regulatory elements of *TaPSKR* genes

**Figure 4:**
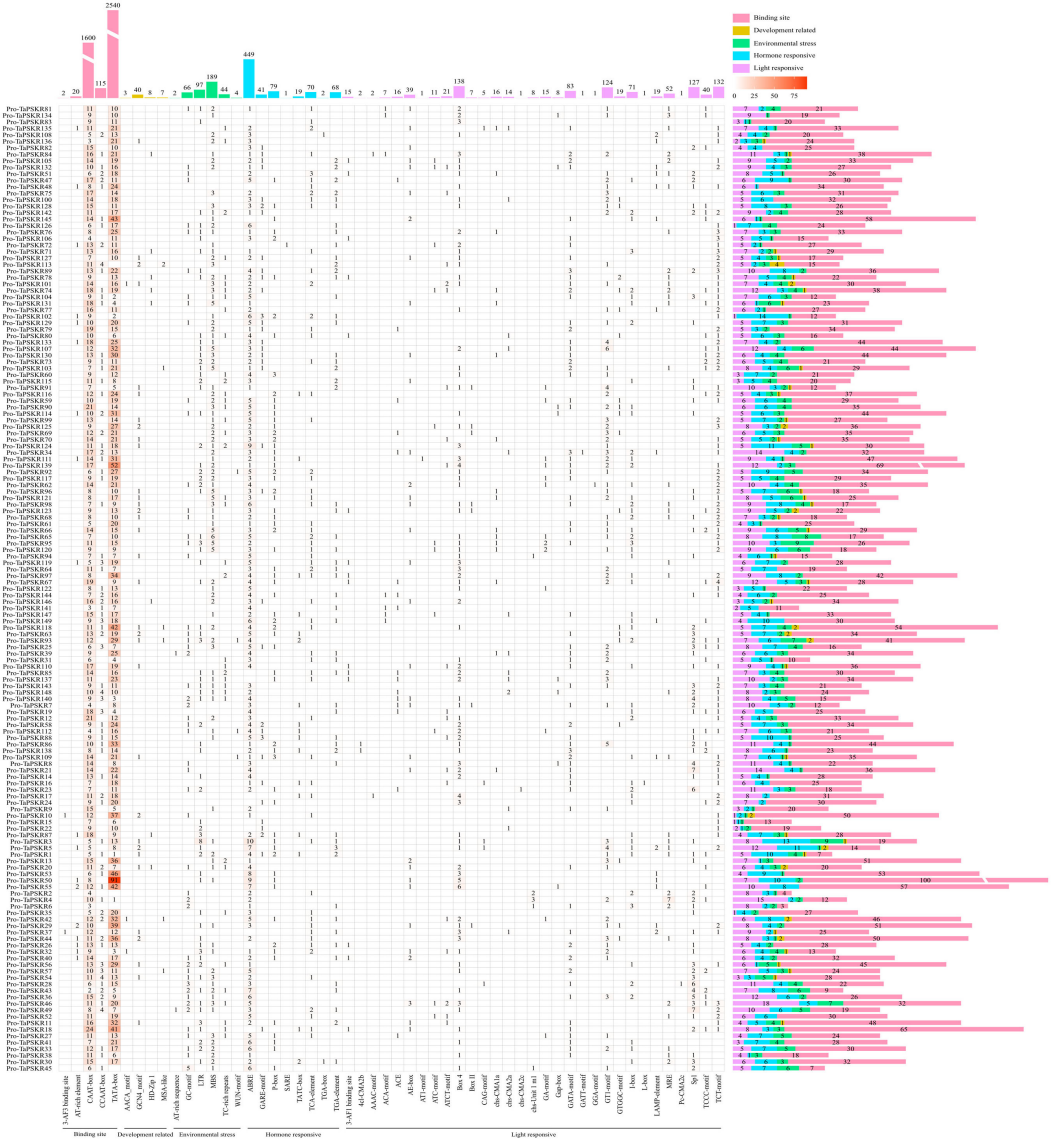
Number and composition of cis-acting regulatory elements in the promotor regions of *TaPSKR* genes. The 2000-bp promoter region of each gene copy is displayed.

Cis-acting elements in the promoter are crucial to regulating gene expression and its function (Lescot et al., 2002). To further predict the bio-function of *TaPSKR* genes, we analyzed the cis-acting elements in the 2-kb promoter region of all *TaPSKR* genes using the online database PlantCARE. As shown in **Figure 4**, a total of 6463 cis-acting were identified across 149 *TaPSKR* genes (**Supplementary Table S6**). These cis-acting elements were classified into 54 types and further categorized into five main functional groups: RNA polymerase-binding site, related to development, environmental stress response, hormone response, and light response (**Figure 4**). Among them, a large number of core elements (4277, 66.18%) were associated with RNA polymerase-binding sites, such as AT-rich elements, CAAT-box, and TAAT-box, indicating that it could enable genes to complete the normal transcription process. Of the cis-elements, 58 (0.9%) responded to development regulation, consistent with a previous study. AACA and GCN4 motifs control the endosperm starch structure in maize (Wu et al., 2015; Liu et al., 2018). Of the cis-elements, 402 (6.22%) were involved in the environmental stress response, of which the MBS and TC-rich repeat motifs in the *AsGRAS* gene family (*GRAS* gene family in *A. sativa*) regulate the salt stress response. A total of 729 (11.28%) cis-elements, such as ABA-responsive elements (ABREs), gibberellin-responsive elements (TCTC-box, P-box, and GARE-motif), auxin-responsive elements (TGA-element), and SA-responsive elements (TCA-element), responded to hormones (Zhang et al., 2024), and 997 (15.43%) cis-elements responded to light. These results suggest that *TaPSKR* genes are involved in plant growth and abiotic stress responses.

### 3.6 Expression profile of *TaPSKR* genes under abiotic stress

To investigate the abiotic stress response of *TaPSKR* genes, we obtained transcriptome data for 149 *TaPSKR* genes from wheat under abiotic stress (such as PEG 6000, salt stress, heat stress, and cold stress) and phytohormone treatments (such as gibberellic acid (GA), jasmonic acid (JA), 6-benzylaminopurine (6BA), ABA, and SA) from WheatOmics. In the tolerant cultivar “Giza 168”, PEG6000 treatment at 2 h and 12 h up-regulated 42 and 32 genes and down-regulated 87 and 102 genes, respectively, whereas in the sensitive cultivar “Gemmiza 10”, 135 TaPSKR genes responded to PEG6000, with 61 up-regulated and 74 down-regulated at 2 h, and 87 up-regulated and 48 down-regulated at 12 h. Heat treatment up-regulated 40 and 42 genes and down-regulated 59 and 71 genes in the thermotolerant “HD2985” and thermosusceptible “HD2329” varieties, respectively. Under salt stress, the salt-sensitive ‘Chinese Spring’ showed up-regulation of 96, 90, and 53 genes and down-regulation of 38, 43, and 80 genes at 6, 12, and 24 h, respectively, while the salt-tolerant “Qingmai 6” exhibited up-regulation of 83, 80, 59, and 50 genes and down-regulation of 51, 57, 80, and 83 genes at 6, 12, 24, and 48 h, respectively. Phytohormones significantly influenced TaPSKR expression: GA and JA treatment at 1 h up-regulated 17 and 45 genes and down-regulated 102 and 74 genes, respectively, while ABA and SA treatment at 3 h up-regulated 24 and 76 genes and down-regulated 76 and 32 genes, respectively. These results suggest that TaPSKR genes are involved in abiotic stress responses and phytohormone regulatory pathways.

### 3.7 Validation of abiotic-responsive *TaPSKR*s through qRT-PCR analysis

**Figure 5:**
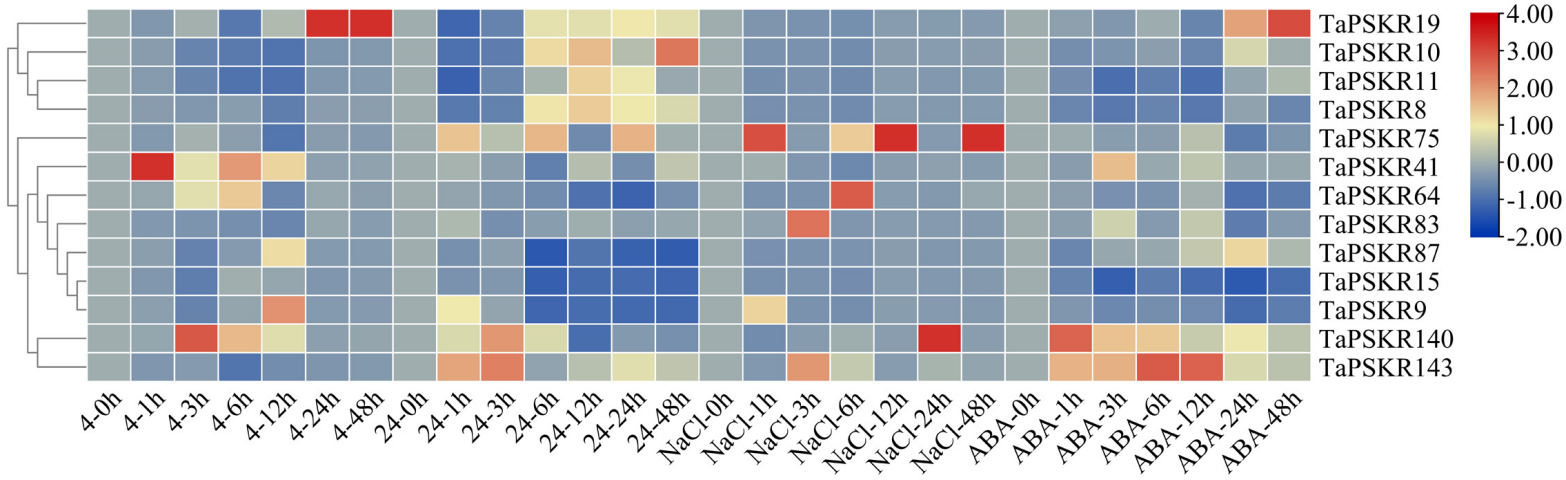
Relative expression levels of 13 randomly selected *TaPSKR* genes under abiotic stress (4°C, 24°C, 200 mM NaCl) and 100 µM ABA conditions using qRT-PCR analysis.

To investigate the abiotic stress response of *TaPSKR* genes, we randomly selected 13 *TaPSKR* genes, namely *TaPSKR8*, *TaPSKR9*, *TaPSKR10*, *TaPSKR11*, *TaPSKR15*, *TaPSKR19*, *TaPSKR41*, *TaPSKR64*, *TaPSKR75*, *TaPSKR83*, *TaPSKR87*, *TaPSKR140*, and *TaPSKR143*, and verified their expression using qRT-PCR under different abiotic stress conditions, such as cold stress, heat stress, salt stress, and ABA treatment (**Figure 5**).

Cold stress disrupts root water uptake and leads to inadequate water supply to the stem, resulting in drought stress (Aroca et al., 2012). Each year, spring frost takes place in March and April at the early booting stage, affecting approximately 85% of the wheat-sown area in the world (Yue et al., 2016). Among these 13 *TaPSKR*s, 7 were less affected by cold stress, the rest of 6 *TaPSKR* genes induced by cold stress (**Figure 5**). Among them, *TaPSKR41* reached the highest expression at 1 h. *TaPSKR140*, *TaPSKR64*, and *TaPSKR9* showed the highest expression at 3, 6, and 12 h, respectively. *TaPSKR19* expression was induced by cold stress at 24 h.

Heat stress increases ROS and lipid peroxidation products in the coleoptile and developing organs and inhibits root and first leaf development at the seedling stage (Savicka and Skute, 2010). Additionally, heat stress alters wheat grain quality, such as grain weight, nutrients, fiber, protein content, and starch granule composition (Akter et al., 2017). In this study, *TaPSKR10* induced by heat at 6 h, and reached the highest expression at 48 h, *TaPSKR143* rapidly response to heat at 1 h, *TaPSKR87*, *TaPSKR15*, and *TaPSKR9* were inhibited by heat stress.

Soil salinity affects approximately 7% of the land mass worldwide and has become a serious environmental stressor increasingly affecting crop production (Yang and Guo, 2018; Hickey et al., 2019). Among the *TaPSKR* genes, *TaPSKR19 TaPSKR10*, *TaPSKR11*, *TaPSKR8*, *TaPSKR41*, *TaPSKR87*, and *TaPSKR15* were less affected by salt stress, while the other 6 *TaPSKR* genes were significantly affected by salt stress. *TaPSKR9* displayed the highest expression at 1 h, and *TaPSKR143* and *TaPSKR83* showed the highest expression at 3 h. The expression of *TaPSKR64*, *TaPSKR75*, and *TaPSKR140* was highest at 6, 12, and 24 h, respectively. The diverse expression patterns of these *TaPSKR*s imply their different roles in the salt stress response.

ABA is a major phytohormone affecting the response to environmental stress and regulating cellular, physiological, and developmental processes (Stone, 2019). Our results indicated that *TaPSKR11*, *TaPSKR8*, *TaPSKR15*, and *TaPSKR9* were down-regulated by ABA. *TaPSKR140*, *TaPSKR41*, *TaPSKR143*, and *TaPSKR19* reached the highest expression under 100 µM ABA condition at 1, 3, 6, and 24 h, respectively.

## 4 Discussion

### 4.1 Systematic identification and comprehensive analysis of *TaPSKR* genes in wheat

Signal transduction is an important way for plants to experience sensory environmental stimuli, which is essential for the regulation of plant growth and development processes and environmental stress responses. PSKR proteins were first isolated in microsomal fractions from carrot cell cultures (*Daucus carota*) (Matsubayashi et al., 2002). A total of 2 and 15 ortholog genes have been identified in *Arabidopsis* and rice, respectively (Matsubayashi et al., 2006; Nagar et al., 2020). Previous studies have shown that *PSKR* genes participate in disease resistance and the drought response, regulating the balance between growth and defense in rice (Yang et al., 2019; Nagar et al., 2022; Harshith et al., 2024), modulating the defense response, and initiating auxin-dependent immunity in tomato (Zhang et al., 2018; Hu et al., 2023). *AtPSKR1* and *AtPSKR2* promote plant growth and callus formation, control pollen tube growth and funicular pollen tube guidance, and regulate immune responses against pathogens (Mosher et al., 2013; Stuhrwohldt et al., 2015; Rodiuc et al., 2016).

In this study, 149 *TaPSKR* genes were identified in the wheat genome (**Supplementary Table S1**). The phylogenetic tree constructed with 149 *TaPSKR*, 2 *AtPSKR*, and 15 *OsPSKR* genes showed that 112 (75.2%) *TaPSKR* genes clustered with *Arabidopsis* and rice genes, forming a distinct branch (**Figure 1**). Clustering suggested that *TaPSKR* genes in wheat had more evolutionary diversification, thereby indicating their functional diversity. Conserved motif analysis of 149 *TaPSKR* genes revealed that the genes grouped within the same branch of the phylogenetic tree had similar conserved motifs (**Figures 2A, B**). This suggests that members with similar conserved motifs may have functional redundancy. Additionally, all *TaPSKR* genes have a single membrane spanning domain (**Figure 2C**), consistent with the structural characteristics of the LRR-RLK family (Sauter, 2015). Among the 149 *TaPSKR* genes, 98 (65.77%) and 51 (34.23%) contained only 1 exon and more than 2 exons, respectively (**Figure 2D**), suggesting that the structural evolution and gene expansion of the *TaPSKR* gene family are relatively conserved (Li et al., 2024).

### 4.2 Expression profile analysis of *TaPSKR* genes in wheat under abiotic stress

**Figure 6:**
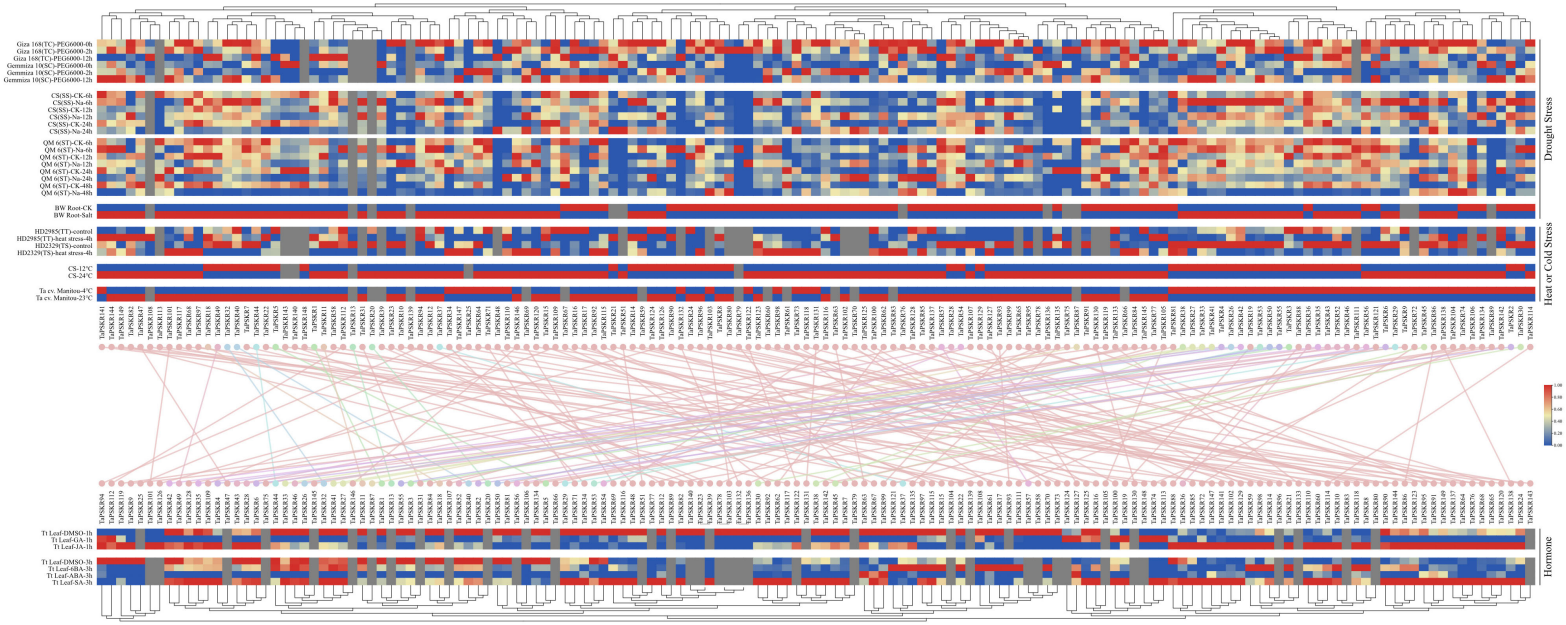
Expression patterns of *TaPSKR* genes under abiotic stress and phytohormone treatment conditions. TC, tolerant cultivar; SC, sensitive cultivar; TT, thermotolerant; TS, thermosusceptible; SS, salt sensitive; ST, salt tolerant; CS, Chinese Spring; QM6, Qing Mai 6; BW, bread wheat; Ta cv., *Triticum aestivum* cv; and Tt, *Triticum turgidum*.

Plant growth and development are finely regulated by external environment factors and internal gene expression levels (Li et al., 2024). To further clarify the bio-functions of *TaPSKR* genes in wheat, we predicted the cis-elements of 149 *TaPSKR* genes. The results revealed that *TaPSKR* genes may be involved in development regulation, environment stress response, hormone response, and light response (**Figure 4**), suggesting that *TaPSKR* genes have diverse functions and play an important role in environment factor response. Additionally, three elements associated with stress responses were identified: MBS, ABRE, and TC-rich repeats. Among these elements, 189 MBS elements and 44 TC-rich repeat elements were identified in 149 *TaPSKR* genes and are involved in multiple environmental stress responses in plants (**Figure 3**). A total of 449 ABREs that participated in the ABA response pathway and abiotic stress responses were identified in the promoters of 149 *TaPSKR* genes. Other response elements, including 41 GARE-motif, 70 P-Box, and 97 LTR endogenous retroviral elements, were also identified. The expression profile results showed that the expression of more than 100 *TaPSKR* genes was influenced by abiotic stress and phytohormone treatments, consistent with the results of our qPCR analysis (**Figures 6** and **5**), indicating that *TaPSKR* genes are involved in a variety of biological processes throughout the life cycle of wheat.

## 5 Conclusion

In this study, 149 *TaPSKR* genes were identified in the wheat genome; among them, 112 *TaPSKR* genes clustered in one group with *AtPSKR* and *OsPSKR* genes. Conserved motif analysis revealed that *TaPSKR* genes within the same branch shared similar conserved motifs, further validating the robustness of the phylogenetic tree. Additionally, each *TaPSKR* gene contained a single transmembrane domain, consistent with the structural characteristics of the LRR-RLK family. Chromosome localization analysis revealed an uneven distribution of the 149 *TaPSKR* genes across 21 chromosomes, with the lowest number on chromosomes 5B and 5D and the highest on chromosome 6A. Cis-element prediction and expression profiling demonstrated that *TaPSKR* genes exhibited complex regulatory mechanisms under various abiotic stress and phytohormone treatments. Our qPCR analysis of 13 randomly selected *TaPSKR* genes supported this result. In summary, our results provide a comprehensive analysis of *TaPSKR* genes in wheat and contribute to molecular breeding for improving abiotic stress response.

## Data Availability

The datasets presented in this study can be found in online repositories. The names of the repository/repositories and accession number(s) can be found in the article/material.

## References

[B1] Ahuja I. Vos Bones A. M. Hall R. D. (2010). Plant molecular stress responses face climate change. Trends Plant Sci. 15, 664–674. doi: 10.1016/j.tplants.2010.08.002, PMID: 20846898

[B2] Akter N. Rafiqul Islam M. Islam R. (2017). Heat stress efects and management in wheat. A review. Agron. Sustain. Dev. 37, 37. doi: 10.1007/s13593-017-0443-9

[B3] Amano Y. Tsubouchi H. Shinohara H. Ogawa M. Matsubayashi Y. (2007). Tyrosine-sulfated glycopeptide involved in cellular proliferation and expansion in Arabidopsis. Proc. Natl. Acad. Sci. U.S.A. 104, 18333–18338. doi: 10.1073/pnas.0706403104, PMID: 17989228 PMC2084343

[B4] Aroca R. Porcel R. Ruiz-Lozano J. M. (2012). Regulation of root water uptake under abiotic stress conditions. J. Exp. Bot. 63, 43–57. doi: 10.1093/jxb/err266, PMID: 21914658

[B5] Balaban M. Moshiri N. Mai U. Jia X. F. Mirarab S. (2019). Tree Cluster: Clustering biological sequences using phylogenetic trees. PloS One 14, e0221068. doi: 10.1371/journal.pone.0221068, PMID: 31437182 PMC6705769

[B6] Burastero O. Cabrera M. Lopez E. D. Defelipe L. A. Arcon J. P. Durán R.. (2022). Specificity and reactivity of mycobacterium tuberculosis serine/threonine kinases pknG and pknB. J. Chem. Inf. modeling 62, 1723–1733. doi: 10.1021/acs.jcim.1c01358, PMID: 35319884

[B7] Chen C. J. Wu Y. Li J. W. Wang X. Zeng Z. H. Xu J.. (2023). TBtools-II: A “one for all, all for one” bioinformatics platform for biological big-data mining. Mol. Plant 16, 1733–1742. doi: 10.1016/j.molp.2023.09.010, PMID: 37740491

[B8] Chitthavalli Y. Harshith A. P. Monoswi C. Ashwin N. Steffi R. Padubidri V. S. (2024). Wound-induced small-peptide-mediated signaling cascade, regulated by OsPSKR, dictates balance between growth and defense in rice. Cell Rep. 43, 114515. doi: 10.1016/j.celrep.2024.114515, PMID: 39003743

[B9] Eun C. H. Ko S. M. Matsubayashi Y. Sakagami Y. Kamada H. (2003). Phytosulfokine-α requires auxin to stimulate carrot non-embryogenic cell proliferation. Plant Physiol. Biochem. 41, 447–452. doi: 10.1016/S0981-9428(03)00052-4

[B10] Godel-Je˛drychowska K. Mackowska K. Kurczynska E. Grzebelus E. (2019). Composition of the reconstituted cell wall in protoplastderived cells of daucus is affected by phytosulfokine (PSK). Int. J. Mol. Sci. 20, 5490. doi: 10.3390/ijms20215490, PMID: 31690047 PMC6862203

[B11] Hallgren J. Konstantinos D. Tsirigos Mads D. P. Armenteros J. J. A. Marcatili P.. (2022). DeepTMHMM predicts alpha and beta transmembrane proteins using deep neural networks. bioRxiv. doi: 10.1101/2022.04.08.487609

[B12] Hanai H. Matsuno T. Yamamoto M. Matsubayashi Y. Kobayashi T. Kamada H.. (2000). A secreted peptide growth factor, phytosulfokine, acting as a stimulatory factor of carrot somatic embryo formation. Plant Cell Physiol. 41, 27–32. doi: 10.1093/pcp/41.1.27, PMID: 10750705

[B13] Harshith C. Y. Pal A. Chakraborty M. Nair A. Raju S. Shuvaprasad P. V. (2024). Wound induced small-peptide mediated signalling cascade regulated by OsPSKR, dictates balance between growth and defense in rice. Cell Rep. 43, 114515. doi: 10.1016/j.celrep.2024.114515, PMID: 39003743

[B14] Hickey L. T. Hafeez N. Robinson H. Jackson S. A. Leal-Bertioli S. C. M. Tester M.. (2019). Breeding crops to feed 10 billion. Nat. Biotechnol. 37, 744–754. doi: 10.1038/s41587-019-0152-9, PMID: 31209375

[B15] Hohmann U. Lau K. Hothorn M. (2017). The structural basis of ligand perception and signal activation by receptor kinases. Annu. Rev. Plant Biol. 68, 109–137. doi: 10.1146/annurev-arplant-042916-040957, PMID: 28125280

[B16] Hu Z. J. Fang H. M. Zhu C. G. Gu S. H. Ding S. T. Yu J. Q.. (2023). Ubiquitylation of PHYTOSULFOKINE RECEPTOR 1 modulates the defense response in tomato. Plant Physiol. 192, 2507–2522. doi: 10.1093/plphys/kiad188, PMID: 36946197 PMC10315268

[B17] Kaufmann C. Sauter M. (2019). Sulfated plant peptide hormones. J. Exp. Bot. 70, 4267–4277. doi: 10.1093/jxb/erz292, PMID: 31231771 PMC6698702

[B18] Kutschmar A. Rzewuski G. Stuhrwohldt N. Beemster G. T. Inze D. Sauter M. (2009). PSK-alpha promotes root growth in Arabidopsis. New Phytol. 181, 820–831. doi: 10.1111/j.1469-8137.2008.02710.x, PMID: 19076296

[B19] Kwezi L. Ruzvidzo O. Wheeler J. I. Govender K. Iacuone S. Thompson P. E.. (2011). The phytosulfokine (PSKα) receptor is capable of guanylate cyclase activity and enabling cyclic GMP-dependent signaling in plants. J. Biol. Chem. 286, 22580–22588. doi: 10.1074/jbc.M110.168823, PMID: 21504901 PMC3121402

[B20] Lescot M. Déhais P. Thijs G. Marchal K. Moreau Y. Van Der Peer Y.. (2002). PlantCARE, a database of plant cis-acting regulatory elements and a portal to tools for in silico analysis of promoter sequences. Nucleic Acids Res. 30, 325–327. doi: 10.1093/nar/30.1.325, PMID: 11752327 PMC99092

[B21] Li Y. Fu Q. Li X. Zhang Q. Zhao Q. Ding Y.. (2024). Exploring the guardian of abiotic stress: Genome-wide identification of the basic helix-loop-helix transcription factor family in Juglans mandshurica. Sci. Hortic. 331, 113154. doi: 10.1016/j.scienta.2024.113154

[B22] Liu X. Li S. Yang W. Mu B. Jiao Y. Zhou X. J.. (2018). Synthesis of seed-specific bidirectional promoters for metabolic engineering of anthocyanin-rich maize. Plant Cell Physiol. 59, 1942–1955. doi: 10.1093/pcp/pcy110, PMID: 29917151

[B23] Loivamaki M. Stu€hrwohldt N. Deeken R. Steffens B. Roitsch T. Hedrich R.. (2010). A role for PSK signaling in wounding and microbial interactions in Arabidopsis. Physiol. Plant 139, 348–357. doi: 10.1111/j.1399-3054.2010.01371.x, PMID: 20403122

[B24] Matsubayashi Y. Ogawa M. Kihara H. Niwa M. Sakagami Y. (2006). Disruption and overexpression of arabidopsis phytosulfokine receptor gene affects cellular longevity and potential for growth. Plant Physiol. 142, 45–53. doi: 10.1104/pp.106.081109, PMID: 16829587 PMC1557600

[B25] Matsubayashi Y. Ogawa M. Morita A. Sakagami Y. (2002). An LRR receptor kinase involved in perception of a peptide plant hormone, phytosulfokine. Science 296, 1470–1472. doi: 10.1126/science.1069607, PMID: 12029134

[B26] Matsubayashi Y. Sakagami Y. (1996). Phytosulfokine, sulfated peptides that induce the proliferation of single mesophyll cells of Asparagus officinalis L. Proc. Natl. Acad. Sci. U.S.A. 93, 7623–7627. doi: 10.1073/pnas.93.15.7623, PMID: 8755525 PMC38796

[B27] Matsubayashi Y. Takagi L. Omura N. Morita A. Sakagami Y. (1999). The endogenous sulfated pentapeptide phytosulfokine-a stimulates tracheary element differentiation of isolated mesophyll cells of zinnia. Plant Physiol. 120, 1043–1048. doi: 10.1104/pp.120.4.1043, PMID: 10444087 PMC59337

[B28] Mosher S. Seybold H. Rodriguez P. Stahl M. Davies K. A. Dayaratne S.. (2013). The tyrosine-sulfated peptide receptors PSKR1 and PSY1R modify the immunity of Arabidopsis to biotrophic and necrotrophic pathogens in an antagonistic manner. Plant J. 73, 469–482. doi: 10.1111/tpj.12050, PMID: 23062058

[B29] Nagar P. Kumar A. Jain M. Kumari S. Mustafiz A. (2020). Genome-wide analysis and transcript profiling of PSKR gene family members in Oryza sativa. PloS One 15, e0236349. doi: 10.1371/journal.pone.0236349, PMID: 32701993 PMC7377467

[B30] Nagar P. Sharma N. Jain M. Sharma G. Prasad M. Mustafiz A. (2022). OsPSKR15, a phytosulfokine receptor from rice enhances abscisic acid response and drought stress tolerance. Physiol. Plantarum 174, e13569. doi: 10.1111/ppl.13569, PMID: 34549425

[B31] Reichardt S. Piepho H.-P. Stintzi A. Schaller A. (2020). Peptide signaling for drought-induced tomato flower drop. Science 367, 1482–1485. doi: 10.1126/science.aaz5641, PMID: 32217727

[B32] Rodiuc N. Barlet X. Hok S. Perfus-Barbeoch L. Allasia V. Engler G.. (2016). Evolutionarily distant pathogens require the Arabidopsis phytosulfokine signalling pathway to establish disease. Plant Cell Environ. 39, 1396–1407. doi: 10.1111/pce.12627, PMID: 26290138

[B33] Sauter M. (2015). Phytosulfokine peptide signalling. J. Exp. Bot. 66, 5161–5169. doi: 10.1093/jxb/erv071, PMID: 25754406

[B34] Savicka M. Skute N. (2010). Efects of high temperature on malondialdehyde content, superoxide production and growth changes in wheat seedlings (Triticum aestivum L.). Ekologija. 56, 26–33. doi: 10.2478/v10055-010-0004-x

[B35] Serrazina S. Martinez M. T. Cano V. Malhó R. Costa R. L. Corredoira E. (2022). Genetic transformation of quercus ilex somatic embryos with a gnk2-like protein that reveals a putative anti-oomycete action. Plants 11, 304. doi: 10.3390/plants11030304, PMID: 35161285 PMC8838351

[B36] Srivastava R. Liu J. X. Howell S. H. (2008). Proteolytic processing of a precursor protein for a growth-promoting peptide by a subtilisin serine protease in Arabidopsis. Plant J. 56, 219–227. doi: 10.1111/j.1365-313X.2008.03598.x, PMID: 18643977 PMC2667306

[B37] Stone S. L. (2019). Role of the ubiquitin proteasome system in plant response to abiotic stress. Int. Rev. Cell Mol. Biol. 343, 65–110. doi: 10.1016/bs.ircmb.2018.05.012, PMID: 30712675

[B38] Stuhrwohldt N. Dahlke R. I. Kutschmar A. Peng X. Sun M. X. Sauter M. (2015). Phytosulfokine peptide signaling controls pollen tube growth and funicular pollen tube guidance in Arabidopsis thaliana. Physiol. Plant 153, 643–653. doi: 10.1111/ppl.12270, PMID: 25174442

[B39] Stührwohldt N. Dahlke R. I. Steffens B. Johnson A. Sauter M. (2011). Phytosulfokine-a controls hypocotyl length and cell expansion in Arabidopsis thaliana through phytosulfokine receptor 1. PloS One 6, e21054. doi: 10.1371/journal.pone.0021054, PMID: 21698171 PMC3116886

[B40] Takahashi F. Shinozaki K. (2019). Long-distance signaling in plant stress response. Curr. Opin. Plant Biol. 47, 106–111. doi: 10.1016/j.pbi.2018.10.006, PMID: 30445314

[B41] Tang Y. Chen H. Deng T. Chang Y. Sun K. T. Ditta A.. (2022). Genome-wide identification and analysis of the GUB_WAK_bind gene family in Gossypium hirsutum. Mol. Biol. Rep. 49, 6405–6413. doi: 10.1007/s11033-022-07449-3, PMID: 35441355

[B42] Wu J. D. Jiang C. P. Zhu H. S. Jiang H. Y. Cheng B. J. Zhu S. W. (2015). Cloning and functional analysis of the promoter of a maize starch synthase III gene (ZmDULL1). Genet. Mol. Res. 14, 5468–5479. doi: 10.4238/2015, PMID: 26125743

[B43] Yang Y. Q. Guo Y. (2018). Elucidating the molecular mechanism smediating plant salt-stress responses. New Phytol. 217, 523–539. doi: 10.1111/nph.14920, PMID: 29205383

[B44] Yang H. Matsubayashi Y. Nakamura K. Sakagami Y. (2001). Diversity of Arabidopsis genes encoding precursors for phytosulfokine, a peptide growth factor. Plant Physiol. 127, 842–851. doi: 10.1104/pp.010452, PMID: 11706167 PMC129256

[B45] Yang W. Zhang B. G. Qi G. H. Shang L. Y. Liu H. F. Ding X.. (2019). Identification of the phytosulfokine receptor 1 (OsPSKR1) confers resistance to bacterial leaf streak in rice. Planta 250, 1603–1612. doi: 10.1007/s00425-019-03238-8, PMID: 31388828

[B46] Yue Y. Zhou Y. Wang J. Ye X. (2016). Assessing wheat frost risk with the support of gis: an approach coupling a growing season meteorological index and a hybrid fuzzy neural network model. Sustainability 8, 1308. doi: 10.3390/su8121308

[B47] Zhang H. Hu Z. Lei C. Zheng C. F. Wang J. Shao S. J.. (2018). A plant phytosulfokine peptide initiates auxin-dependent immunity through cytosolic ca2+ Signaling in tomato. Plant Cell 30, 652–667. doi: 10.1105/tpc.17.00537, PMID: 29511053 PMC5894845

[B48] Zhang Y. Peng Y. Zhang H. Gao Q. Song F. Mo F. (2024). Genome-Wide Identification of APX Gene Family in Citrus maxima and Expression Analysis at Different Postharvest Preservation Times. Genes 15, 911. doi: 10.3390/genes15070911, PMID: 39062690 PMC11276291

